# Evidence-Based Bibliometric Analysis of Research on Silver Diamine Fluoride Use in Dentistry

**DOI:** 10.1155/2021/9917408

**Published:** 2021-09-29

**Authors:** Syed Saad B. Qasim, Dena Ali, Abdul Samad Khan, Shafiq Ur Rehman, Abid Iqbal, Jagan Kumar Baskaradoss

**Affiliations:** ^1^Department of Bioclinical Sciences, Faculty of Dentistry, Health Sciences Centre, Kuwait University, Safat, P.O.Box 24923, Kuwait 13110; ^2^Department of General Dental Practice, Faculty of Dentistry, Health Sciences of Centre, Kuwait University, Safat, P.O.Box 24923, Kuwait 13110; ^3^Department of Restorative Dental Sciences, College of Dentistry, Imam Abdulrahman Bin Faisal University, Dammam 31441, Saudi Arabia; ^4^Institute of Information Management, University of the Punjab, Quaid-e-Azam Campus, Lahore, Pakistan; ^5^Central Library, Prince Sultan University, Rafha Street, Riyadh, Saudi Arabia; ^6^Department of Developmental and Preventive Sciences, Faculty of Dentistry, Health Science Centre, Kuwait University, Safat, P.O.Box 24923, Kuwait 13110

## Abstract

**Background:**

This bibliometric analysis is aimed at reviewing the research pattern on the use of silver diamine fluoride (SDF) in dentistry using various citation metrics.

**Methods:**

A well-curated search was conducted on Elsevier's Scopus database for the relevant literature on SDF published between 1969 and 2021. Bibliographic information such as information related to citations, bibliographic data, abstracts, keywords, and other relevant information was extracted using different combinations of keywords (“silver diamine fluoride” OR “Silver Diamine Fluorides” OR “Diamine Fluoride” OR “Silver Fluoride”). Analysis and visualization of the selected documents and related data were performed using various tools and software including MS Excel, MS Access, Bibexcel, VOS viewer, Biblioshiny, and Gephi. VOS Viewer was utilized for the Graph Modeling Language (GML) to generate graphical representations of the data. Furthermore, network graphs were generated to assess the various associations between research themes, countries, organizations, authors, journals, and citations.

**Results:**

The initial search yielded 662 documents, of which 410 were chosen for analysis. 252 records were deemed irrelevant. The chosen records consisted of journal articles (*n* = 351), conference papers (*n* = 14), book chapters (*n* = 1), and review articles (*n* = 44). The results showed that there was an upward trend in the research on SDF, and a substantial increase was observed in the citation index after 2014. Researchers from the United States of America, Hong Kong, and Japan were the top contributors, with organizations and authors from the Faculty of Dentistry, University of Hong Kong, leading the way in citations and productivity.

**Conclusion:**

The bibliometric analysis provides valuable information regarding the total number of publications on SDF and their citation details. It also identifies the leading countries and organizations involved in the research on SDF and provides a comprehensive analysis of the research trends related to SDF.

## 1. Introduction

According to the Global Burden of Diseases, untreated caries in primary dentition are the 10th most common condition currently affecting humans [1]. This has been attributed to several factors such as limited or lack of access to oral health care services in the community, improper exposure to fluoride, and increased consumption of sugary food and beverages. These findings are indicative of the fact that existing therapeutic strategies have not been able to inhibit dental caries. Hence, there is a need to investigate new or alternative approaches to control cariostatic activity in pediatric patients [2]. Silver salts have been extensively mentioned in the current literature for their ability to elicit a potent antimicrobial response in young dentition [3–5].

Dr Nishino and Yamaga are considered the pioneers in this field for successfully adapting ammoniacal silver fluoride for arresting dental caries in patients [6]. After the initial experimentation, the existing formulation of these salts was combined with fluoride and silver ions to form silver diamine fluoride (SDF). SDF has been proven to be useful in the management of many dental conditions. It is indicated for managing early childhood caries [7], erosive tooth wear, tooth sensitivity as well as desensitization of non-carious tooth or hypersensitive dentin [8], and molar incisor hypomineralization [9]. SDF is a colorless ammonia solution made of silver and fluoride ions. Since the neutral silver fluoride exists in an unstable form, it is usually mixed with water containing ammonia to give it a more stable structure [10]. The diamine-silver ion is a complex ion with two ammonia molecules connected to a silver ion. The fluoride in the SDF has a well-proven track record for exaggerating the remineralization of enamel and dentine [11], whereas the silver has been proven to have a strong antibacterial activity [12].

In 2014, the Food and Drug Administration (FDA) in the USA cleared SDF to be marketed as a class II medical device that could be used to treat tooth sensitivity. However, it has also being used offlabel to treat and prevent dental caries [13]. The American Dental Association (ADA) Council on Scientific Affairs recommends the biannual application of a 38% SDF solution as the treatment of choice for nonrestorative management of advanced cavitated carious lesions on any coronal surface of permanent or primary teeth [14]. The need for agents like SDF is perhaps best understood in terms of the World Health Organization (WHO) Millennium Development Goals for Health [15]. The proposed path to achieving these goals involves laying more emphasis on emergency care, prevention, and developing newer cost-effective interventions [16]. To achieve these goals, simpler technologies would be required to “scale up” dental services and to improve the access to oral health care that is cost-effective and affordable. Furthermore, all preventive interventions would need to be built on firm evidence-based foundations. The use of SDF in dentistry for the prevention and treatment of dental caries has gained great momentum over the past few years [4]. Systematic reviews conducted in the past on SDF have reported its use not only in the prevention of dental caries but also in arresting carious lesions in paediatric as well as geriatric patients. Its effect on root caries prevention and arrest is also being explored by different research clusters across the globe [17–19].

While SDF has been routinely used in Japan for over 80 years [20], its use is now increasing at the global level. Consequently, there has been growing interest among researchers in investigating the use of SDF in dentistry. The last decade, in particular, has seen exponential growth in the research on SDF. However, a comprehensive analysis of this published research is lacking. There is a need to analyze the published articles on the topic both quantitatively and qualitatively. A comprehensive systemic analysis would help understand the current research on the topic and highlight any gaps that need to be filled. A bibliometric analysis is a quantitative measure that aids to assess the scholarly literature by analysing various aspects of research articles such as citation analysis, publication counts, institutional affiliations, and geographical distribution [21]. Bibliometric indicators aid in measuring, assessing, and comparing the research output and impact of individual researchers, groups, and organizations. Different databases have been used in such analyses such as Scopus (Elsevier, Netherlands), Web of Science (Clarivate Analytics, USA), and Medline (National Library of Medicine (NLM), USA).

This investigation is aimed at reviewing the research progress on silver diamine fluoride in dentistry by using a bibliometric approach. The objective was to analyze research productivity, publication and citation structure, leading countries and institutions, most productive authors, and leading journals, major themes, keyword co-occurrences, bibliographic coupling of countries, authors, and journals from 1969 to 2021 in the field of SDF. It is hoped that this systemic bibliometric analysis would help overcome hurdles amongst research clusters across the globe working on investigating the clinical potential of SDF in dentistry and provide researchers with a comprehensive overview of the current state of affairs regarding research on SDF in dentistry.

## 2. Materials and Methods

A bibliometric analysis (a quantitative method) of the academic literature, published on the topic “silver diamine fluoride use in dentistry,” was performed in this study. Different variables such as the total citations (TC), total publications (TP), publishing year (PY), journal's impact factor (IF), authorship patterns, and the *h*-index were identified. The most productive authors, journals, countries, and organizations were also identified from the retrieved data.

### 2.1. Database and Bibliographic Information Selection

Scopus is a well-known, curated, and high-quality bibliographic database. It was selected for data extraction on publications on “silver diamine fluoride use in dentistry.” Bibliographic information related to citations, abstracts, keywords, and other relevant bibliographic information was extracted.

### 2.2. Search Query

Bibliographic data on silver diamine fluoride use in dentistry was searched by the following query, using the advanced search interface of Scopus: “Article Title, Abstract, Keywords.” Search terms were combined by the Boolean operator “OR”: “*silver diamine fluoride*” *OR* “*Silver Diamine Fluorides*” *OR* “*Diamine Fluoride*” *OR* “*Silver Fluoride*.”

### 2.3. Date of Data Extraction

The abovementioned database was searched at the Imam Abdul Rehman Bin Faisal University, Saudi Arabia, on January 13, 2021. The initial search yielded a total of 662 documents.

### 2.4. Inclusion/Exclusion Criteria

**Figure 1:**
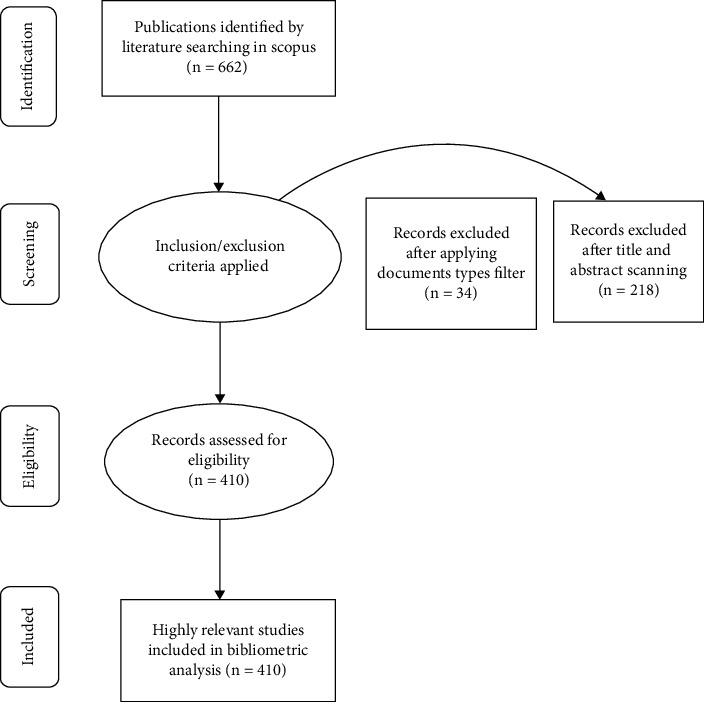
Four phase flow charts of data extraction and filtration process of Silver diamine fluoride publications.

A comprehensive inclusion and exclusion criteria, as shown in Figure 1, were applied to extract the most relevant and comprehensive data. The search was limited to articles, review articles, conference papers, books, and book chapters. No language, geographical, or time (date) filters were applied. However, editorials and short surveys were excluded. The initial search yielded 662 documents. Each record was then screened for relevance and eligibility, and 252 irrelevant records were excluded. Conclusively, 410 records consisting of journal articles (*n* = 351), conference papers (*n* = 14), book chapters (*n* = 1), and review articles (*n* = 44) were selected for the bibliometric analysis. The publication date of the selected records ranged from 1969 to 2021. The records were finally exported into MS Excel. To ensure the correctness of data, this process was repeated by two of the authors of the study.

### 2.5. Data Analysis and Visualization

The analyses and visualization of the 410 selected documents and their related data were performed using various tools and software including MS Excel (v16.0), MS Access (v16.0), Bibexcel, VOS viewer (version 1.6.15), Biblioshiny (version 2.0), and Gephi (version 0.9.2). The VOSViwere software was used to obtain the Graph Modeling Language (GML) files from the raw data. These GML files were then exported into Gephi for network analysis and network visualization. The bibliometric indicators with respect to citation, documents, sources, authors, organizations, and countries were also studied. Lastly, the publication and citation trends, authorship patterns, bibliographic couplings (journals, countries, and authors), co-occurrences of keywords, cocitation network of authors, and thematic evolution were visualized with the help of the aforementioned tools and software.

## 3. Results

### 3.1. Research on Silver Diamine Fluoride (1969-2021)

**Figure 2:**
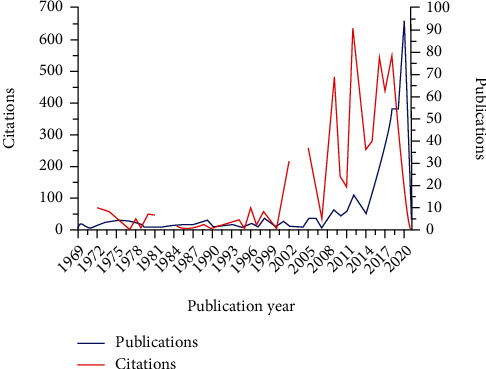
Publications and citation trends in silver diamine fluoride over a period of 1969-2021. This break in the citation growth is because the publication did not receive any citation during that period. (The figure was generated using MS Excel.)

Figure 2 shows the annual research productivity in SDF research in terms of publications and citations. It can be seen that the first two publications on the topic were published in the year 1969 and collectively received 31 citations. Since then, the research productivity has progressively increased. There was significant activity during the second decade of the current century (2011-2020) with 317 publications published during the time (77% of the total publications). Furthermore, during the same period, these 317 publications received 3642 citations (62% of the total citations). The maximum number of publications (*N* = 94) occurred in the year 2020. On the other hand, the 16 publications that were published in the year 2012 were the most highly cited (635 citations) among all.

### 3.2. Publishing and Citation Structure of Silver Diamine Fluoride

Table 1 displays the annual publishing and citation details of SDF research. It can be seen in the table that approximately 80% (*n* = 328) of the total (*n* = 410) publications had received 5835 citations. 50 out of the 54 publications published in the year 2019 received 315 citations. 48 articles out of the 55 cited publications (55 TP) in the year 2018 received 556 citations. The average citations per publication (C/P) and average citations per cited publication (C/CP) in the year 2002 were 214. The year 2009 had a C/P and C/CP of 53.6, followed by the year 2005 with C/P and C/CP of 51.6.

Table 1 shows the annual citation structure of silver diamine fluoride research between 1969 and 2021. It also shows the total publications (TP), number of cited publications (NCP), total citations (TC), citation impact [publication] (C/P), and citation impact [cited publication] (C/CP).

### 3.3. Leading Countries and Institutions

Analysis was conducted to determine the countries and institutes leading the way with the highest number of publications. The citations received by these publications and their citation impact were also calculated as shown in Table 2. The countries with the highest number of publications were (1) United States (*n* = 194), (2) Hong Kong (*n* = 79), and (3) Japan (*n* = 49). Publications from the United States of America had been cited 1249 times with a citation impact of 13.15. Despite a lower publication volume as compared to the USA, Hong Kong led the way in way in citations with a total of 2364 citations and a citation impact of 29.92 for its 79 publications. Indonesia, the only Islamic country in the top ten most productive list, had the lowest citation impact (2.36) with only 14 publications and 33 citations.

As for the leading institutions, the University of Hong Kong topped the list of organizations with the most publications in SDF research. As shown in Table 2, there were 94 publications from the University of Hong Kong, which had received the highest number of citations (2773) and had a citations impact of 29.50. The Tokushima University produced nine publications, which received 137 citations with a citations impact of 15.22. The University of Adelaide published 10 highly cited publications (358 citations) with 35.8 average citations per paper, followed by the Universidade de Pernambuco with 13 publications, 434 citations, and a citation impact of 33.38. The results show that 5 out of 10 organizations in this list published 160 (75.5%) documents that had received 3774 (78.2%) citations.

Table 2 lists the top countries and organizations in silver diamine fluoride research between 1969 and 2021. It also shows total publications (TP), total citations (TC) associated with these publications, and citations impact (CI).

### 3.4. Most Productive Authors

The data was sorted based on total publications from various authors to identify the top 10 most productive authors. The total citations, citation impact, and *h*-index were also calculated to highlight their respective influence in this area of research.  Table 3 ranks the most prolific authors involved in silver diamine fluoride research. Lo from the University of Hong Kong produced the highest number of publications (62), which were cited 2141 times and had an average of 34.53 citations per publication and an *h*-index of 26, whereas Crystal from New York University College of Dentistry, United States of America, was ranked tenth with 11 publications, 195 citations, and an *h*-index of 7. Rosenblatt (Universidade De Pernambuco, Brazil) had a citations impact of 36 for his 12 publications (432 citations).

### 3.5. Leading Journals

Table 4 presents the top 10 most preferred journals in SDF research. These ten journals published 137 publications (33.41% of 410 total publications) and accumulated 3269 citations (56% of 5835 total citations). In terms of the citations counts, the Journal of Dental Research, the Journal of Dentistry, and the Australian Dental Journal emerged as the top sources with 1211, 802, and 371 citations for their 19, 27, and 20 publications, respectively.

Three journals from this list are in the 1^st^ quartile, two in the 2^nd^ quartile, three in the 3^rd^, and two in the 4^th^ quartile. There were no nonimpact factor journals in this list. Most of the journals belonged to the United States of America (6) and England (3), while only one was published in Switzerland.

This table lists the top journals on silver diamine fluoride between 1969 and 2021. It also shows the journal's impact factor (IF), its quartile (*Q*), and publisher. The country of origin is given in the last column.

### 3.6. Bibliographic Coupling of Journals

**Figure 3:**
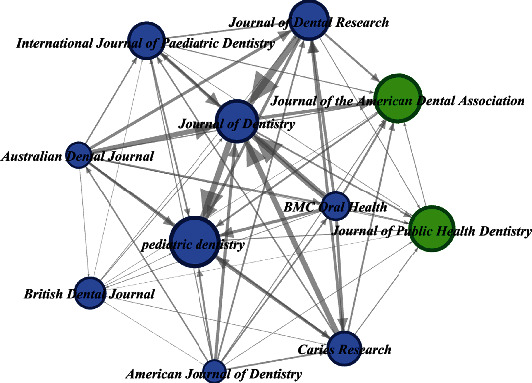
Bibliographic coupling of a total of 152 journals. The minimum number of doc = 7 and 11 meets the threshold. (generated using Gephi). Arrows pointing towards Journal of Dentistry shows a stronger relationship as compared with other journals.

Network graphs enable visualization of the bibliographic coupling of journals. These graphs usually consist of edges (the connections, here, they represent the publications), nodes (the entities, here, they represent the journals), and arrows that show the directed network type (in our case, all networks were directed and based on referrals). The size and color of the nodes (journals) and arrows depend on the data associated with them. Subject matter commonality among journals is measured by the bibliographic coupling of journals. Journals are bibliographically coupled if they refer to a common third publication.  Figure 3 explains this phenomenon among journals on SDF research. In our analysis, journals with a minimum of seven documents were included. 11 of the 152 journals met this criterion. Journals in SDF research were classified into two clusters and visualized using the Gephi package. Journals with the highest bibliographic coupling activity included the Journal of Dentistry (26 documents, 800 citations, and total link strength of 4950); the Australian Dental Journal (20 documents, 371 citations, and total link strength of 2101); and the Journal of Dental Research (19 documents, 1211 citations, and total link strength of 2822).

### 3.7. Major Themes in Silver Diamine Fluoride

Table 5 lists the twenty-five themes found in SDF research. 590 publications have been classified under the 25 themes. They had received a total of 10991 citations. SDF emerged as the top theme with 145 publications and 1785 citations (12.31 average citations per publication (C/P), followed by dental caries with 74 publications and 1447citations with 19.55 C/P.

The 3 major themes that emerged in the selected 3 time periods were as follows:

P1 (1969-2010)silver fluoride (12), dental caries (7), dentine (6)

P2 (2011-2015)silver diamine fluoride (23), dental caries (12), fluoride (11)

P3 (2016-2021)silver diamine fluoride (117), dental caries (55), caries (32)

This table lists the main themes included in the silver diamine fluoride research and shows the total publications (TP), total citations (TC), and citation impact (C/P) associated with these themes. P1, P2, and P3 (1969-2010, 2011-2015, 2016-2021) present the count of the number of publications for each period.

### 3.8. Keyword Co-Occurrences in Silver Diamine Fluoride

**Figure 4:**
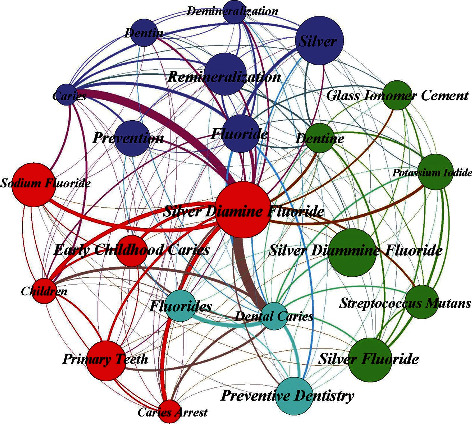
Keywords co-occurrences with minimum co-occurrences of 12 times. 22 meet the threshold (generated using Gephi). Arrows pointing towards silver diamine fluoride and dental caries showing a strong relationship. Nodes represent keywords, and the line thickness (edge weight) represents the degree of connection.

Figure 4 depicts the keyword co-occurrences in SDF research. Keywords that had a minimum of 12 co-occurrences or more were included. 22 keywords met this criterion. Figure 4 shows that the top five keywords were silver diamine fluoride, dental caries, caries, fluoride, and children with 147, 74, 39, 36, and 19 weights of occurrences, respectively. Keywords (demineralization, dentin, glass ionomer cement, and preventive dentistry) showed minimal co-occurrences (12) in this group. Closely related keywords were then classified into four clusters, and quantitative network indicators were used to describe the connections between those clusters. Silver diamine fluoride (the keyword with the highest occurrences) can be seen in cluster 4 along with children, primary teeth, caries arrest, early childhood caries, and sodium fluoride.

### 3.9. Bibliographic Coupling of Countries

**Figure 5:**
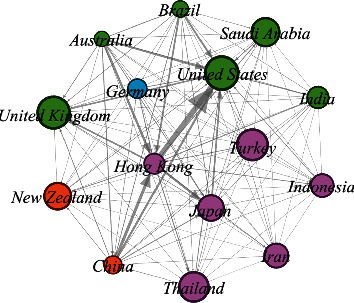
Bibliographic coupling of countries that published a minimum of 8 documents. 15 countries, out of the 65 countries in the study, met the threshold (generated using Gephi).

Figure 5 displays the bibliographic coupling of countries in SDF research. Countries with a minimum of 8 documents or more were included; 15 of the 65 countries met the criterion. Countries highly involved in bibliographic coupling included the United States of America (95 documents, 1249 citations, and 38175 total link strength); Hong Kong (79 documents, 2364 citations, and 44031 total link strength); and Japan (49 documents, 675 citations, and 15076 total link strength). Countries can be seen grouped into four clusters based on their close relations in content and connections as described by using quantitative network indicators. The United States of America, the country with the highest research output on SDF (Table 2), can be seen in cluster 2 along with United Kingdom, Saudi Arabia, India, Brazil, and Australia. Germany is in cluster 4.

### 3.10. Bibliographic Coupling of Authors

**Figure 6:**
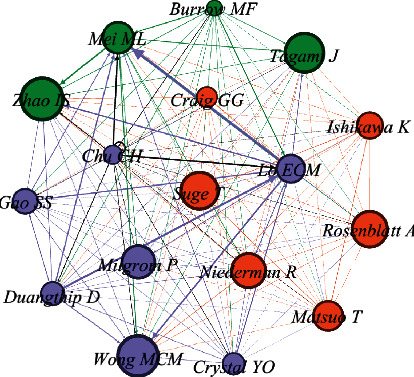
Bibliographic coupling of authors with minimum 8 documents of an author, 17, meets the threshold (generated using Gephi).

Figure 6 presents the bibliographic coupling of authors involved in SDF research. Authors with a minimum of 8 documents were included, and 17 meet the criterion. Authors with the highest bibliographic coupling activity included Lo (53 publications, 1760 citations, and 33397 total link strength); Chu (46 documents, 1393 citations, and 30497 total link strength); and Mei (30 documents, 800 citations, and 24760 total link strength). Authors were further classified under three different clusters. There were 7 authors in clusters 1 and 6 and in clusters 2 and 4 authors in cluster 3.

### 3.11. Cocitation Network of Authors

**Figure 7:**
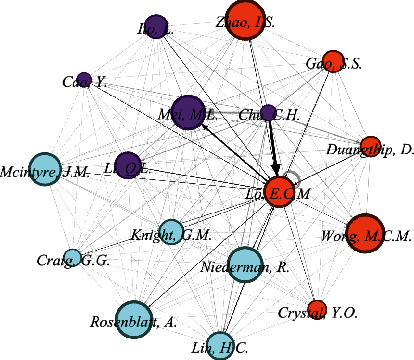
Cocitation network of authors with minimum citations = 115. 17 met the threshold (generated using Gephi). Strong relationship denoted with black arrow pointing from Chu C.H and Lo E.C.M.

Figure 7 presents the cocitation network of authors in SDF research between 1969 and 2021. Authors with 115 or more citations were included; 17 authors meet the criterion. It shows that top five authors are Lo, Chu, Mei, Duangthip, and Lin with weight age (citations) of 1304, 1100, 536, 274, and 267, respectively. Authors are grouped into clusters, and quantitative network indicators connect these clusters. There are 3 total clusters of authors: cluster 1 (5 authors), 2 (6 authors), and 3 (6 authors).

### 3.12. Authorship Pattern

**Figure 8:**
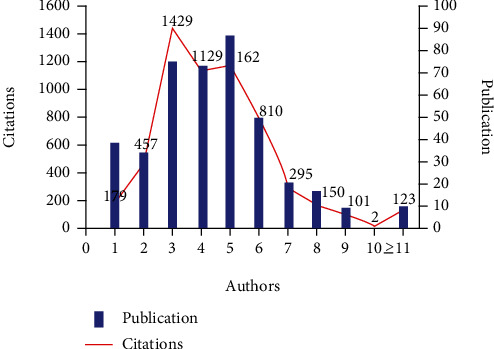
Authorship pattern of silver diamine fluoride (generated using MS Excel), showing citations in the *X*-axis, number of authors in the *Y*-axis, and publicaitons in the *Z*-axis.

In this study, the authorship patterns focused on the authors' characteristics and the degree of collaboration among authors involved in a specific publication under study. The total number of authors per document was calculated in the study.  Figure 8 represents the results of the authorship pattern analysis. The top three authorship patterns were five authors (87 publications), three authors (75 publications), and four authors (73 publications). Only two documents with 10 authors each were found. However, there were 10 documents with more than 11 contributors. The highly cited documents with 1429, 1162, and 1129 citations were authored by 3, 5, and 4 authors, respectively.

### 3.13. Three Field Plot (Countries, Keywords, and Journals)

**Figure 9:**
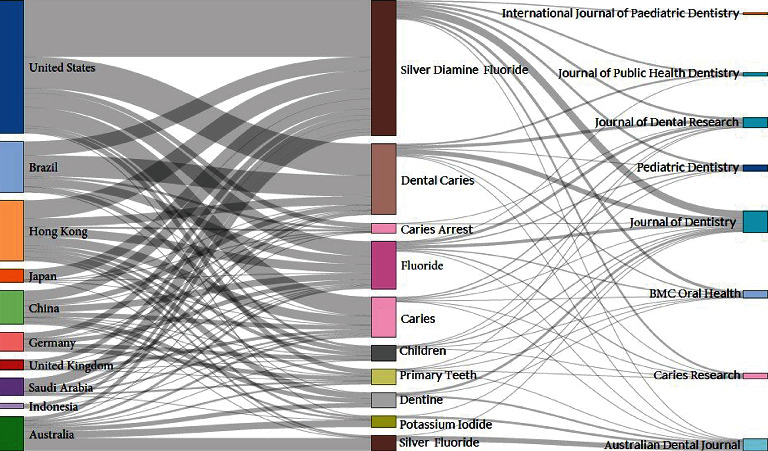
Three field plot of countries (left), keywords (middle), and journals (right) (generated using Biblioshiny).

Figure 9 demonstrates the three field plot of the relationship among countries, keywords, and journals. It shows that five countries (USA, Hong Kong, Brazil, China, and Australia) had published SDF literature using four main keywords (silver diamine fluoride, dental caries, caries, and fluoride). Research on SDF originated from these countries using these keywords was published in the following journals and had a strong relationship: Australian Dental Journal, Caries Research, Journal of Dental Research, Journal of Dentistry, and pediatric dentistry.

### 3.14. Thematic Evolution Map of Keywords

**Figure 10:**
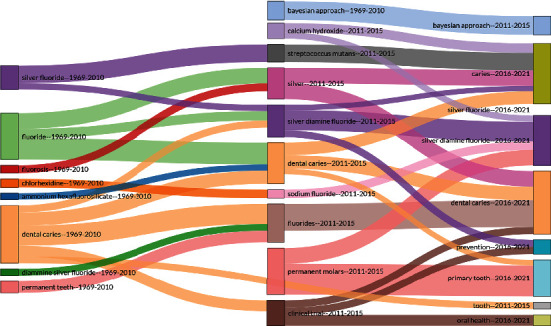
Thematic evolution map of keywords from 1969 to 2021 with respect to silver diamine fluoride research. Usage and emergence of keywords related to this study are highlighted (generated using Biblioshiny).

Figure 10 reveals the evolution of keywords in three different stages; 1969-2010, 2011-2015, and 2016-2021. It can be observed that during the 1969-2010 period, the SDF themes dental caries and flouride had a high number of citations. This shows that these two themes had a high impact during this period. During the 2011-2015 period, the permanent molars, flouride, dental caires, silver diamine flouride, and silver themes received much attention from the researchers in the field of SDF.

Lastly, the documents published during the 2016-2021 period received high citations for the caries, silver, silver diamine flouride, and dental caries themes. On the other hand, themes such as tooth and oral health did not garner much attention during this period.

## 4. Discussion

This bibliometric analysis is the first of its kind as it aimed to identify and quantitatively evaluate the scientific research articles published on the use of SDF in dentistry during the last five decades.

### 4.1. Yearly Trend of Publications

The yearly citation and publication analysis of the research articles revealed that the first paper related to the use of ammonical silver fluoride for arresting dental caries was published in 1969 [6] in The Journal of Osaka University Dental School. It has received 29 citations. The other significant publication on SDF has been cited 214 times and was published by Chu et al. in the Journal of Dental Research (Q1) in 2002. Chu et al. claimed that SDF was effective in arresting dentine caries of primary teeth in preschool children. The trend of publishing on SDF has been slowly growing and has seen exponential growth after the year 2014.

### 4.2. Leading Countries and Institutions

The results of the analysis have highlighted that the United States of America (USA), Hong Kong, and Japan were the top three most productive countries, with the USA having the highest number of publications and citations. This could be attributed to the existence of the National Institute of Dental and Craniofacial Research (NIDCR) in the USA which actively provides funding to both private and public sectors for dental research. Hong Kong had the second largest number of publications and citations after the United States of America. As for the most productive organizations, the University of Hong Kong led the way followed by the University of Washington, Seattle, and Tokyo Medical and Dental University, Japan. The Indonesian University was also amongst the top 10 most productive organizations, highlighting the promotion of research in this domain among the South East Asian countries. Furthermore, the domination of Hong Kong in the most productive countries and institution lists could be attributed to the presence of several different research clusters in the same institution having a similar field of interest [22–24]. Consequently, 5 of the top 10 highly cited authors, namely, Mei [22], Chu [24], and Lo [23], and Burrow M [25], were affiliated with The University of Hong Kong.

### 4.3. Leading Journals and Bibliographic Coupling of Journals

Three out of the top 10 journals were enlisted in the Q1 ranking of the Web of Science database. These were equally distributed in between Elsevier (England), Sage (USA), and the American Dental Association (USA). The highly cited papers by Chu et al. [24] and Rosenblatt et al. [4] were both published in the Journal of Dental Research (Q1) by Sage Publishing Inc. (USA). Interestingly, all of the top 10 journals have been published either in England, the United States of America, or Switzerland. This is indicative of the trend that the developed countries have been making significant contributions within this field of research. Furthermore, they publish manuscripts in the English Language only, as were the records selected in our study.

### 4.4. Major Themes in SDF

The systemic analysis also revealed the major themes in SDF research and the prevalent trends in publications on this specialty. Keywords aided researchers in conducting a targeted search for the relevant articles related to SDF in this study. The analysis of the more frequently used keywords helps investigators to target published research articles using the correct terms. The findings of the analysis showed that silver diamine fluoride (2011-2021) was a frequently used keyword in the last decade alongside dental caries (1969-2021) which has been used since the very beginning. This could be attributed to the fact that the Food and Drug Administration (FDA) approved the use of SDF for treating caries in 2014 and granted it breakthrough therapy status to treat severe early childhood caries in 2016 [26]. A similar trend was observed in the analysis of the number of publications, citations, and research collaboration. It should be noted that there have been reports of discoloration of dentition associated with the use of SDF, and alternative formulations are now readily available with potassium iodide which help with the reduction in the discoloration [27–29]. Dental caries has been the most commonly used keyword from 1969 to 2021, most likely due to the common application of SDF in different carious lesions in teeth, i.e, in enamel, dentine, or root caries [30].

### 4.5. Keyword Co-Occurrences in SDF

The keywords had a specific trend among the publication chosen for analysis. As a rule, keyword co-occurrence networks are based on extracted keywords from the titles and abstracts of publications or even from the author generated keyword list. Keywords are said to co-occur if they both occur in the same title/abstract or citation context. Moreover, the distance between them is almost inversely proportional to the similarity of the keywords. Therefore, keywords with a higher rate of relevance are generally found to be closer to each other [31]. It was found that SDF, Caries, Fluoride, Remineralization, and Silver Fluoride have been the frequently used keywords. However, keywords related to staining were noticeably absent. In addition, the use of antistaining agents such as potassium iodide has not been highlighted in keywords so far [32, 33].

### 4.6. Bibliographic Coupling of Countries and Authors

The bibliographic coupling analysis is done to help reveal how a single item of reference is shared between two documents [34]. This analysis, when performed for countries, revealed that the United States of America, Hong Kong, and Japan were amongst the top performers and had the maximum number of connections. Similarly, regarding the bibliographic coupling of authors, authors from various academic institutions in Hong Kong had the highest number of bibliographical coupling in SDF research. Other authors at the forefront of this activity were from Australia and Brazil and Japan and Saudi Arabia. However, authors from the Faculty of Dentistry, University of Hong Kong, had significant contributions in this regard. They were from different departments of the university such as the Department of Restorative Dental Sciences and the Department of Applied Oral Sciences and Community Dental Care [22]. This could be attributed to the fact that SDF has been used to treat carious lesions since the 1960s in multiple countries in that region including Japan and China as well as in other countries such as Australia, Brazil, and Argentina. On the other hand, SDF has only been approved for dental use fairly recently by the Food and Drug Administration (FDA) in the United States of America [35].

### 4.7. Cocitation Network of Authors

In a bibliographic analysis, cocitation network analysis of authors helps to analyze the underlying specialties in a field in terms of the groups of authors who have been cited together in the relevant literature. Moreover, it also delivers insights into how authors, as domain experts, perceive the interconnectivity between published works [36]. Authors from the Faculty of Dentistry, the University of Hong Kong, Lo ECM [37], Chu, Mei, Zhao, and Gao [7, 35], have been identified as major contributors in research collaborations. This field of research appears to have been dominated by this cluster so far, and we would likely see more collaborative projects in the future from this cluster.

### 4.8. Authorship Pattern, Three Field Plots, and Thematic Evaluation Maps of Keywords

The findings in this study showed that the trend of publications had a strong link with the selection of keywords. It is important to use relevant and appropriate keywords which can be easily searched, keeping in mind that different groups of readers may use various terms to describe the same information. The three-factor relationship showed that “silver diamine fluoride” and “dental caries” keywords were mainly used by authors from the USA, Brazil, Hong Kong, China, Australia, Japan, Saudi Arabia, and Germany. Interestingly, the keyword “dental caries” is a somewhat generic term while “silver diamine fluoride” is a very specific term related to the topic. The term “silver fluoride” was mainly seen in publications originating from Australia which had been mainly published in the Australian Dental Journal. It was also observed that studies originating from the USA had been published in different journals, with the majority being published in Pediatric Dentistry (the official publication of the American Academy of Pediatric Dentistry), the Journal of Dental Research, the American Journal of Dentistry, and the Journal of the American Dental Association. On the other hand, most British researchers had mainly published in the British Dental Journal and BMC Oral Health. This particular trend shows that the researchers tended to focus on journals being published in their own respective countries.

Furthermore, it was noted that the term “silver diamine fluoride” was also used in articles from Japan, China, the USA, Brazil, and Hong Kong but not in those from Australia. Keywords associated with the methods of application of SDF and its properties such as “antibacterial” and “remineralization” were used less frequently.

In the thematic evaluation of the data, it was found that keywords such as “dental caries,” “silver fluoride,” and “fluoride” have been used throughout the last 52 years. Similarly, “silver fluoride” has been used frequently during the 1969-2010 period. However, the term “sliver diamine fluoride” has only been used since 2011, and in the last five years, it has been used along with keywords “caries” and “prevention.” The use of the keyword “silver diamine fluoride” after 2011 might be due to its approval as a Class II medical device for the treatment of dental hypersensitivity by the US Food and Drug Administration. In 2009, WHO classified SDF as an “effective, efficient, equitable and safe caries-preventative agent” [4]. However, the term “dental hypersensitivity” was not a commonly used keyword in this study.

The results of this study highlight that there has been significant variation in terms of the nature and size of the collaborating teams in SDF research. The analysis of the authorship pattern and network structure confirmed that a high number of publications had multiple authors (3, 4, or 5). This can be related to the frequent collaborations among various institutes, countries, and researchers to expedite the development of this area of research. Notably, a very high number of authors (10 or 11) had a very low number of publications. It could be because nowadays most journals discourage a high number of authors (more than 6) per publication to circumvent the trend of guest authorship.

### 4.9. Limitations and Future Research Directions

This study was limited by the use of a single database for the collection of bibliographic data. Other databases such as the Web of Science, PubMed, and Google Scholar were not taken into account. Furthermore, the factor of self-citation was not analyzed as there is currently no methodology available to do so. Regardless of its limitations, this study on silver diamine fluoride could aid future investigators in identifying possible avenues for research and filling the identified gaps in research. Also, it would help them identify the most influential countries, universities, authors, journals, and keywords while researching the topic and looking for collaborations.

Future areas of interest for researchers could include expansion of the search to other databases, investigating the use of nanosilver sodium fluoride [38] in dentistry and its clinical applications as compared to SDF, the staining potential of SDF, and novel formulations that can minimize or neutralize the staining caused by SDF.

## 5. Conclusions

This bibliometric analysis provides valuable information on the total number of publications on SDF and their citation details during the 1969 to 2021 period. It has shown that there was an overall upward trend in publishing on the topic with a significant increase in the number of publications after 2014. It has also identified the leading countries and organizations involved in the research on SDF and the research trends related to it. Even though the use of silver diamine fluoride has become prevalent in the last few decades, only the last few years have seen significant scientific publications. It is hoped that this study would allow budding and existing researchers to imagine and build future scenarios for scientific collaborations for research on the use of SDF in dentistry.

## Figures and Tables

**Table 1:** Citation structure of silver diamine fluoride publications between 1969 and 2021.

PY	TP	TC	NCP	C/P	C/CP
1969	2	31	2	15.5	15.5
1970	1	0	0	0.0	0.0
1971	1	0	0	0.0	0.0
1972	2	69	2	34.5	34.5
1974	4	55	4	13.8	13.8
1976	4	15	3	3.8	5.0
1977	3	4	3	1.3	1.3
1978	3	35	3	11.7	11.7
1979	1	4	4	4.0	1.0
1980	1	50	1	50.0	50.0
1981	1	44	1	44.0	44.0
1982	1	0	0	0.0	0.0
1984	2	9	2	4.5	4.5
1985	2	2	1	1.0	2.0
1987	2	5	2	2.5	2.5
1989	4	17	3	4.3	5.7
1990	1	1	1	1.0	1.0
1993	2	20	2	10.0	10.0
1994	1	34	1	34.0	34.0
1995	1	2	1	2.0	2.0
1996	3	68	3	22.7	22.7
1997	1	17	1	17.0	17.0
1998	6	57	5	9.5	11.4
1999	2	40	2	20.0	20.0
2000	1	1	1	1.0	1.0
2001	4	127	4	31.8	31.8
2002	1	214	1	214.0	214.0
2004	1	0	0	0.0	0.0
2005	5	258	5	51.6	51.6
2006	5	124	5	24.8	24.8
2007	1	36	1	36.0	36.0
2008	4	205	4	51.3	51.3
2009	9	482	9	53.6	53.6
2010	6	166	5	27.7	33.2
2011	8	137	8	17.1	17.1
2012	16	635	15	39.7	42.3
2013	11	405	11	36.8	36.8
2014	7	247	7	35.3	35.3
2015	14	277	14	19.8	19.8
2016	23	549	21	23.9	26.1
2017	35	434	32	12.4	13.6
2018	55	556	48	10.1	11.6
2019	54	315	50	5.8	6.3
2020	94	87	39	0.9	2.2
2021	5	1	1	0.2	1.0
Total	410	5835	328	1000.9	1019

**(a):** 

Top 10 countries				
Rank	Country	TP	TC	CI
1	United States	95	1249	13.15
2	Hong Kong	79	2364	29.92
3	Japan	49	675	13.78
4	Australia	37	803	21.70
5	Brazil	37	659	17.81
6	China	31	776	25.03
7	United Kingdom	21	125	5.95
8	India	18	99	5.50
9	Indonesia	14	33	2.36
10	Germany	13	99	7.62

**(b):** 

Top 10 organizations				
Rank	Organization	TP	TC	CI
1	The University of Hong Kong	94	2773	29.50
2	University of Washington, Seattle	24	328	13.67
3	Tokyo Medical and Dental University	15	206	13.73
4	University of Indonesia	14	33	2.36
5	Universidade de Pernambuco	13	434	33.38
6	New York University College of Dentistry	12	131	10.92
7	The University of Western Australia	11	102	9.27
8	The University of Adelaide	10	358	35.80
9	Anhui Medical University	10	325	32.50
10	Tokushima University	9	137	15.22

**Table 3:** The top 10 most influential authors.

Rank	Authors	Affiliation	Country	TP	TC	C/P	*h*-index
1	Lo, E.C.M.	The University of Hong Kong	Hong Kong	62	2141	34.53	26
2	Chu, C.H.	The University of Hong Kong	Hong Kong	58	1819	31.36	25
3	Mei, M.L.	University of Otago, Dunedin,	New Zealand	30	800	26.67	16
4	Duangthip, D.	The University of Hong Kong	Hong Kong	18	437	24.28	11
5	Wong, M.C.M.	The University of Hong Kong	Hong Kong	18	284	15.78	8
6	Zhao, I.S.	Shenzhen University, Shenzhen,	China	14	252	18.00	9
7	Milgrom, P.	University of Washington	United States	13	190	14.62	7
8	Burrow, M.F.	The University of Hong Kong	Hong Kong	12	156	13.00	6
9	Rosenblatt, A.	Universidade De Pernambuco	Brazil	12	432	36.00	8
10	Crystal, Y.O.	New York University College of Dentistry	United States	11	195	17.73	7

This table itemizes the top authors on silver diamine fluoride between 1969 and 2021 and shows the institution and countries of these authors. Total publications (TP), total citations (TC), citations impact (C/P), and *h*-indices (*h*) of these authors are also calculated.

**Table 4:** Leading journals in silver diamine fluoride research.

Rank	Journal	TP	TC	IF	*Q*	Publisher	Country
1	Journal of Dentistry	27	802	3.242	1	Elsevier	England
2	Australian Dental Journal	20	371	1.401	3	Wiley	United States
3	Journal of Dental Research	19	1211	4.914	1	Sage	United States
4	BMC Oral Health	14	243	1.911	2	BMC	England
5	Pediatric Dentistry	14	204	1.594	3	American Academy of Pediatric Dentistry	United States
6	Caries Research	12	174	2.186	2	Karger Publishers	Switzerland
7	American Journal of Dentistry	10	56	0.957	4	Mosher & Linder, Inc	United States
8	Journal of Public Health Dentistry	7	46	1.743	3	Wiley	United States
9	Journal of The American Dental Association	7	136	2.803	1	Amer Dental Assoc	United States
10	British Dental Journal	7	26	1.306	4	Springer Nature	England

**Table 5:** Main themes found in the silver diamine fluoride publications between 1969 and 2021.

SR.	Themes	TP	TC	C/P	P1	P2	P3
					1969-2010	2011-2015	2016-2021
1.	Silver diamine fluoride	145	1785	12.31	5	23	117
2.	Dental caries	74	1447	19.55	7	12	55
3.	Caries	38	831	21.87	1	5	32
4.	Fluoride	36	976	27.11	5	11	20
5.	Children	19	492	25.89	1	4	14
6.	Silver fluoride	18	368	20.44	12	1	5
7.	Dentine	17	466	27.41	6	5	6
8.	Fluorides	17	558	32.82	4	3	10
9.	Potassium iodide	16	297	18.56	5	0	11
10.	Primary teeth	16	253	15.81	0	1	15
11.	Silver diamine fluoride	16	123	7.69	1	1	14
12.	Caries arrest	15	320	21.33	1	2	12
13.	Remineralization	15	168	11.20	0	2	13
14.	Early childhood caries	14	130	9.29	1	0	13
15.	Prevention	14	323	23.07	1	3	10
16.	Silver	14	432	30.86	2	2	10
17.	Streptococcus mutans	14	193	13.79	3	3	8
18.	Sodium fluoride	13	178	13.69	2	2	9
19.	Demineralization	12	147	12.25	1	1	10
20.	Dentin	12	165	13.75	0	1	11
21.	Glass ionomer cement	12	268	22.33	5	1	6
22.	Preventive dentistry	12	587	48.92	4	3	5
23.	Oral health	11	143	13.00	0	2	9
24.	Pediatric dentistry	10	170	17.00	1	0	9
25.	Root caries	10	171	17.10	0	2	8
